# Accelerometry-Based Assessment of Physical Activity in Older Adults During COVID-19 Pandemic

**DOI:** 10.1093/geroni/igab046.3652

**Published:** 2021-12-17

**Authors:** Renoa Choudhury, Ladda Thiamwong, Oscar Garcia, Rui Xie, Jeffrey Stout, Joon-Hyuk Park

**Affiliations:** 1 University of Central Florida, Orlando, Florida, United States; 2 College of Nursing, University of Central Florida, Orlando, Florida, United States

## Abstract

COVID-19 pandemic has caused a severely detrimental effect on the physical, psychological, and functional well-being of older adults by forcing them to limit their social activities. This study investigates the amount and intensity of daily physical activity (PA) in older adults, living under the social distancing guidelines during COVID-19 pandemic. In this cross-sectional study, 124 community-dwelling older adults (Age:60–96 years, mean:75±7.14) were recruited via word-of–mouth and key person approach between March 2021-August 2021. Participants completed an online survey on COVID-19 questionnaires and wore an ActiGraph GT9X accelerometer on their non-dominant wrist for consecutive 7 days in free-living conditions. Euclidean Norm Minus One cut-points were used to estimate the total time spent in sedentary behavior (SB), light PA (LPA) and moderate-to-vigorous PA (MVPA). Results showed that, 7% had COVID-positive, 55% perceived moderate severity of COVID in their area, 31% reported fear of COVID, and 14.5% were afraid of losing their life to COVID. On average, participants obtained 12.43±2.1 hours/day of SB, 3.47±1.05 hours/day of LPA and 42.71±29.71 minutes/day of MVPA. MVPA minutes/day was significantly higher (P = 0.006) in participants aged 60-85 years than those aged 85+ years (45.38 minutes/day vs 14.25 minutes/day). When age-adjusted data was compared to pre-COVID-19 studies, we found COVID-19 pandemic had negatively impacted the physical activity level in older adults (29.33% decrease in MVPA and 39.2% increase in SB). These findings can be useful in developing guidelines and/or interventions to promote physical activity and healthy aging among older adults, particularly those in social isolation.

